# Late onset Alzheimer’s disease genetics implicates microglial pathways in disease risk

**DOI:** 10.1186/s13024-017-0184-x

**Published:** 2017-05-26

**Authors:** Anastasia G. Efthymiou, Alison M. Goate

**Affiliations:** Department of Neuroscience, Ronald M. Loeb Center for Alzheimer’s disease, Icahn School of Medicine at Mount Sinai, 1425 Madison Ave, New York, NY 10029 USA

**Keywords:** Alzheimer’s disease, Genetics, Microglia, Myeloid

## Abstract

Alzheimer’s disease (AD) is a highly heritable complex disease with no current effective prevention or treatment. The majority of drugs developed for AD focus on the amyloid cascade hypothesis, which implicates Aß plaques as a causal factor in the disease. However, it is possible that other underexplored disease-associated pathways may be more fruitful targets for drug development. Findings from gene network analyses implicate immune networks as being enriched in AD; many of the genes in these networks fall within genomic regions that contain common and rare variants that are associated with increased risk of developing AD. Of these genes, several (including *CR1, SPI1, the MS4As, TREM2, ABCA7*, *CD33,* and *INPP5D*) are expressed by microglia, the resident immune cells of the brain. We summarize the gene network and genetics findings that implicate that these microglial genes are involved in AD, as well as several studies that have looked at the expression and function of these genes in microglia and in the context of AD. We propose that these genes are contributing to AD in a non-Aß-dependent fashion.

## Background

AD is a chronic, incurable, neurodegenerative disease that affects an estimated 5.4 million people in the United States [1]. It is a complex disease that is highly heritable, and several genes have been found to be associated with risk for developing AD. Early onset AD (EOAD), diagnosed in individuals who are under the age of 65 years, accounts for a small percentage of all AD cases (5–10%). Mutations in three genes, *APP* [2], *PSEN1* [3, 4], and *PSEN2* [5, 6], are associated with autosomal dominant AD (ADAD), a subset of EOAD [7]. This has led to the prevailing mechanistic theory for AD: the amyloid cascade hypothesis [8]. However, no therapies that target this pathway have yet been successful in preventing the development of or ameliorating the effects of this disease in humans. Late onset AD (LOAD) shares the same clinical and pathological features of early onset AD, but is diagnosed in individuals who are over the age of 65 years. The most important genetic risk factor for the development of LOAD and sporadic EOAD is presence of the E4 variant of the *APOE* gene (*APOE4*) [9, 10].

Clinically, AD manifests as a gradual and unrelenting decline of memory [11]. Patients are diagnosed based on a variety of cognitive factors, but confirmation of an AD diagnosis can only be made by observing the underlying neuropathology. As such, people who are suffering from AD may be misdiagnosed with other forms of dementia, and vice versa, thereby reducing the power of clinical trials and the effectiveness of treatment.

Memory loss in AD is accompanied by neuronal loss and the accumulation of extracellular Aß plaques and intracellular neurofibrillary tangles of hyperphosphorylated Tau. In ADAD, the accumulation of Aß plaques results from improper processing of APP, which may be a consequence of mutations within the *APP* gene itself, or in associated factors such as *PSEN1* and *PSEN2*. In LOAD and sporadic AD, there is no evidence of improper APP processing that leads to amyloid accumulation. However, there is evidence that the clearance of Aß is disrupted [12].

Research on LOAD has shown that there is a slow accumulation of Aß in the brain for up to twenty years before the manifestation of any cognitive symptoms. It is possible that this prodromal state of AD may be an effective therapeutic target, and limiting the accumulation of Aß may halt the development of further symptoms. Biomarkers for AD, including amyloid imaging and monitoring cerebrospinal fluid levels for Aß and Tau, are currently used to inform diagnosis. However, in a clinical setting, they can only be utilized after cognitive symptoms have already become apparent. Understanding how these biomarkers exist in non-demented individuals will be important to inform our future diagnosis and treatment of disease (e.g. Do all non-demented individuals with amyloid accumulation develop clinical AD and if so over what time period?). Similarly, accumulation of Tau is characteristic of other neurodegenerative diseases and is also observed in elderly non-demented individuals. Understanding the natural course of this accumulation will be important to advise therapy.

Current knowledge about the neuropathologic and genetic information informs current theories about the development and progression of AD. However, there are still no good therapies for its prevention or treatment. A possible explanation is that there may be other pathways that are also disrupted in AD, which are not accounted for in the current version of the amyloid cascade hypothesis, and which could serve as more efficient therapeutic targets and/or should be part of combination therapies.

Early descriptions of AD include additional neuropathological features, including gliosis and neuroinflammation, the contributions of which are under investigation. The presence of these features implicates microglia, the resident immune cell type in the brain, in AD pathology. However, these observations do not indicate, for example, whether microglia accumulation around plaques is a consequence or cause of disease [13, 14]. Recently, genetic studies have implicated microglial function as a causal factor in LOAD, as opposed to merely a biological response [15].

**Fig. 1:**
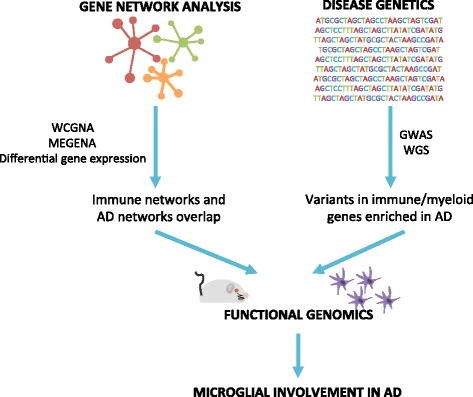
Framework of support for microglia involvement in LOAD. Findings from gene network analysis and disease genetics converge to show enriched immune and myeloid contributions in AD. These findings are verified through functional genomics using cell and animal models to determine the contribution of these genes to LOAD pathology

In recent years, genome-wide association studies (GWAS), whole genome sequencing (WGS), and gene-expression network analysis have uncovered gene networks and common and rare genetic variants that are associated with LOAD. These genes are involved in previously understudied pathways, including cholesterol metabolism, endocytosis, and innate immunity [16]. The GWAS findings and gene expression network analysis both implicate immune and microglial networks as important players in the development and progression of AD (Fig. 1). However, the exact contribution of microglia is not well understood. Genetic and early functional studies suggest that microglia may directly contribute to AD pathology [13]. This review highlights several genes identified in these studies that confer risk for AD and are involved in innate immunity. By looking at the role of microglia in AD, we may uncover novel therapeutic targets to help treat this disease.

## AD GWAS studies

AD genetics implicate microglial and immune genes as being important players in the development and progression of disease. These investigations include genome-wide association studies (GWAS), which must then be validated through functional genomics approaches and large-scale sequencing projects. In this section we will discuss the genes identified in AD GWAS studies and how these genes support the involvement of microglia.

### Choose wisely: population sample and size in GWAS studies

GWAS are used to simultaneously examine millions of genetic variants in tens or hundreds of thousands of individuals to identify variants that are associated with a particular trait or disease state. The most common study design for AD GWAS is a comparison of unrelated disease cases with unrelated elderly non-demented controls from the same population. Importantly, GWAS identify regions of the genome (loci) that exhibit association with a trait rather than specific genes. Indeed, some loci may contain a single gene while others can contain many genes (Table 1). The challenge is to then fine-map these loci to identify specific genes that contain causal functional variants and to understand the mechanisms of pathogenesis leading to increased or decreased risk for disease.

**Table 1:** AD-risk loci identified through genome-wide analyses (GWAS and GWAX) [24, 25]

SNP	Chr	Reported gene	Additional genes in locus
rs6656401	1	*CR1*	
rs6733839	2	*BIN1*	
rs35349669	2	*INPP5D*	*NGEF, NEU2*
rs190982	5	*MEF2C*	*LOC645323*, *MIR9-2*
rs2074612^a^	5	*SCIMP*	*ZNF594, RABEP1, USP6*
rs9271192	6	*HLA-DRB5–* *HLA-DRB1*	*C6orf10, HLA-DRB5, HLA-DRB6, HLA-DRB1, HLA-DQA1, HCG23, BTNL2, HLA-DRA, HLA-DQB1, HLA-DQA2, HLA-DQB2, HLA-DOB, TAP1, TAP2, PSMB8, PSMB9*
rs10948363	6	*CD2AP*	*GPR111, GPR115*
rs2718058	7	*NME8*	*GPR141*
rs1476679	7	*ZCWPW1*	*TRIM4, GJC3, AZGP1, AZGP1P1, ZKSCAN1, ZSCAN21, ZNF3, COPS6, MCM7, C7orf59, GPC2, STAG3, GATS, PVRIG, SPDYE3, PMS2P1, PILRA, PILRB, PPP1R35, MEPCE, NYAP1, SAP25, AGFG2, LRCH4, ZASP, LRCH4, FBXO24, PCOLCE-AS1*
rs11771145	7	*EPHA1*	*CLCN1, FAM131B, ZYX, TAS2R60, LOC285965, TAS2R41*
rs28834970	8	*PTK2B*	*STMN4, TRIM35, CHRNA2, EPHX2*
rs9331896	8	*CLU*	*PTK2B, CHRNA2, EPHX2, SCARA3, CCDC25, ESCO2, PBK*
rs7920721^a^	10	*ECHDC3*	
rs10838725	11	*CELF1*	*ARFGAP2, PACSIN3, DDB2, ACP2, MADD, MYBPC3, SPI1, SLC39A13, PSMC3, RAPSN, PTPMT1, KBTBD4, NDUFS3, C1QTNF4, MTCH2, AGBL2, FNBP4, NUP160*
rs983392	11	*MS4A6A*	*PLAC1L, MS4A3, MS4A2, MS4A4A, MS4A6E*
rs10792832	11	*PICALM*	*CCDC83*
rs11218343	11	*SORL1*	
rs17125944	14	*FERMT2*	*ERO1L, PSMC6, STYX, GNPNAT1*
rs10498633	14	*SLC24A4*	*RIN3*
rs59685680^a^	15	*SPPL2A*	*TRPM7, USP50*
rs77493189^a^	17	*HBEGF*	*HBEGF*
rs4147929	19	*ABCA7*	*CFD, MED16, PRTN3, R3HDM4, KISS1R, ELANE, ARID3A, TMEM259, GRIN3B, WDR18, HMHA1, CNN2, POLR2E, SBNO2, GPX4, ATP5D, MIDN, CIRBP, EFNA2*
rs7274581	20	*CASS4*	*CSTF1, RTFDC1, GCNT7, FAM209A, FAM209B*

Disease-associated SNPs that have reached genome-wide significance for each locus are labeled, along with their chromosome, the closest gene (that which is reported) and additional genes within the locus

^a^identified in GWAX

GWAS are most frequently performed in populations of European origin, but can be very informative if conducted in multiple populations due to the differences in linkage-disequilibrium (LD) structure across different groups. Some loci may show stronger effects in specific populations (e.g. *ABCA7* in African Americans [17]), which can aid in identification of functional variants, but generally LD differences help to narrow the region of association, particularly in African ancestry populations [18].

The importance of sample size cannot be overestimated in GWAS. Meta-analysis of multiple GWAS is commonly used to increase sample size and greatly increase the number of loci discovered and validated as associated with disease [19]. In AD, GWAS with samples of 5000 cases or less could only identify the *APOE* locus as a region associated with disease [20]. Studies with sample sizes of 6000 identified three loci, which were then replicated in larger cohorts [21–23]. Once sample size increased to near 10,000 cases, several loci could be identified and replicated. In 2013 a meta-analyses of >70,000 samples conducted by the International Genomics of Alzheimer’s Project (IGAP) in Caucasian populations has identified more than 20 genetic loci that are associated with AD [24]. In contrast, a GWAS conducted in an African American population with less than 2000 cases identified only one locus outside of the APOE region (*ABCA7)* to be associated with AD [17].

Sample size can also compensate for restricted phenotype data, as shown in a recent study using family history of AD as a proxy in the UK Biobank dataset, which identified several novel loci associated with AD [25]. This study, which included over 100,000 individuals, was described as a genome-wide association study by proxy (GWAX) and replaced disease cases with their first-degree relatives. Despite the absence of a disease phenotype, this study was still able to replicate the main findings from earlier smaller studies and identify four novel loci due to the number of samples involved. This removes an important barrier for identifying disease-associated loci through genetic screens by increasing the population from which “cases” can be obtained.

### Know what you’re getting: GWAS results and limitations

It is common practice to display GWAS data in Manhattan plots, with chromosome location on the x-axis and degree of association (as –log_10_(*p*-value)) on the y-axis, with each data-point representing one single nucleotide polymorphism (SNP). SNPs whose *p*-values are lower than the genome-wide significance cutoff of 10^−8^ are considered significant, and often labeled with the gene that is closest to that locus. However, it is important to note that this labeling based on *proximity* of the most significant SNP to a gene in that locus does not necessarily mean that the disease-associated SNP has any functional interaction or influence on that gene. In fact, it is possible that the genes affected by these variants are many kilobases away. Additionally, it is hard to distinguish SNPs with high LD, indicating the possibility that the actual SNP that confers risk for developing the disease is not the one determined to be most significant by GWAS. Table 1 displays 25 AD-risk loci that have been detected through GWAS meta-analyses, along with all genes within the nearest recombination hotspots of the most significant SNP in that locus. Additional studies will have to be conducted to conclude which gene is affected by that variant.

### Validating GWAS results with functional genomics

The limitations presented by GWAS necessitate functional validation of all target loci in order to determine which gene is affected by these variants or other variants in LD, the effect of the variant on gene expression and function, and subsequently, the effect of the variant on the disease state. This can be done by first fine-mapping the region using computational approaches, which integrate information including transcription factor binding sites, chromatin state, DNA methylation, splicing, and gene expression, to prioritize SNPs and determine which is most likely to have a functional effect and on what gene. Fine-mapping should be followed by in vitro and in vivo studies that seek to validate the SNP-gene pair and to determine their effects in appropriate models. Several examples of functional genomics have been conducted for putative AD-risk genes, including *SPI1* [26] and *CD33* [27], discussed below.

Many of the loci identified through GWAS do not contain genes that are highly expressed in whole brain tissues, impeding our ability to fine-map these loci and assess their contribution to disease. It is possible that this is a result of the heterogeneity of cell types in these samples. Microglia share a similar developmental lineage, gene expression pattern, and function with other peripheral immune cells of the myeloid lineage. Because these gene expression signatures are conserved, there are two ways of interpreting these data. First; microglia contribute to the etiology of AD, and second; peripheral myeloid cells, such as monocytes and macrophages, could be contributing alone, or in combination with microglia, to the observed immune effect. The connection of these cells to other peripheral myeloid cells can be exploited to learn more about the underlying neuropathology of LOAD. Previous work by Raj et al. profiled purified monocytes from a young and healthy population and looked at their gene expression patterns for enrichment across multiple diseases [28]. They found that AD susceptibility alleles are enriched among expression quantitative trait loci (eQTLs) in monocytes but not in T cells. This suggests that monocytes could be used as a proxy to examine the effects of microglia in AD.

### AD-risk genes in microglia

The importance of validating GWAS hits with functional genomics cannot be understated. However, despite the absence of thorough and complete functional studies, integrating information from multiple sources can provide evidence in support of specific genes and pathways. Below, we describe several genes that are present within AD-risk loci, are expressed in microglia, associate with other microglial or immune genes through co-expression analysis, and have previously been shown to be associated with AD in human or animal studies. We also discuss several other genes that have been detected in GWAS and may be involved in these same pathways, which still need to be supported with additional evidence.

### Genes identified through common variants in GWAS

#### CR1

Complement receptor 1 (CR1) is a glycoprotein expressed on immune-related cells, including microglia. Its encoding gene, *CR1*, is located on chromosome 1q32 and is represented by four different alleles that vary in size, transcript, and frequency among populations [29]. The *CR1* locus has been identified through GWAS as a risk factor for AD [21], and complement factors are highly expressed in AD brain [30, 31]. The AD-risk SNP identified in IGAP, rs6656401, falls within an intronic region of *CR1*, with SNPs in high LD spanning the entire length of *CR1* [32]. Another LOAD-associated SNP, rs3818361 ((r^2^ > 0.8 with rs6656401), was reported to be strongly associated with disease in *APOE4* carriers [21]. Both SNPs are associated with low *CR1* expression in brain tissue and neurons, and higher expression in monocytes [33].

CR1 plays a major role in regulating immune activation through the complement cascade and acts as the main receptor for the complement protein C3b [34]. The complement cascade is known to mediate microglial activity, including pruning of synapses [35]. Aβ has been shown to activate the complement system through an association with C1q, which binds to CR1 [36–39]. Increased CR1 is associated with more active microglia, and blocking CR1 impedes microglial ability to phagocytose Aβ [40]. However, given that CR1 is expressed in several cell types in the brain and in peripheral immune cells it is unclear whether the genetic effects of CR1 on AD are mediated through functional effects in a specific cell type or through all cells in which it is expressed [29].

#### CELF1/SPI1

The IGAP meta-analysis identified the SNP rs10838725 to be a risk factor for AD, and this variant is present within an intronic region of the gene *CELF1* [24]. However, LD in this region is extensive, and there are multiple genes that could be affected by the identified AD risk SNP (Table 1). One of these genes, *SPI1*, has been implicated in LOAD through network analysis [41]. Additional studies suggest that PU.1, a transcription factor encoded by the gene *SPI1*, is a central hub in an AD gene network and is associated with AD pathology [41, 42].

*SPI1* is highly expressed in immune cells, macrophages, and microglia, which share a developmental lineage [43, 44]. Further analyses of GWAS data and fine mapping of this locus by Huang et al., indicate that *SPI1* is the gene affected in this locus [26]. Fine-mapping identified 5 variants associated with AD and *SPI1* expression in myeloid cells, including macrophages and monocytes. These variants are located within PU.1 binding sites or a miR-569 binding site within the 3′ UTR of *SPI1*, consistent with potential effects on gene expression. Colocalization analyses of this GWAS locus with variants that influence gene expression suggest that *SPI1*, rather than other nearby genes including *CELF1* and *MYBPC3*, is most likely the gene in this locus contributing to LOAD [26].

The PU.1 cistrome, which includes transcription factor binding sites throughout the entire genome, is also enriched in AD [26]. Chromatin immunoprecipitation (ChIP)-Seq data from myeloid cells shows that PU.1 binds *TYROBP*, *MS4As*, *INPP5D*, *TREM2*, and *CD33*, suggesting that it regulates their gene expression. These recent findings regarding *SPI1* emphasize the importance of verifying genetic targets through a variety of functional genomics approaches.

#### MS4A family

The membrane-spanning 4-domain subfamily A (*MS4A*) gene cluster is present on chromosome 11q12 and includes eighteen genes spanning approximately 600kb [45–47]. Multiple GWAS in European and Asian populations have implicated several genes within the MS4A cluster in AD, including *MS4A4A*, *MS4A4E*, *MS4A6A*, and *MS4A6E* [23, 48–51]. The IGAP meta-analysis identified rs983392, upstream of *MS4A6A* and downstream of *MS4A2*, as an AD-risk SNP that is associated with reduced LOAD risk. This SNP is associated with chromatin marks that confer low transcription in brain tissues and neuronal cells, and is associated with enhancers and active transcription sites in peripheral primary human monocytes [33]. Another SNP, rs670139, lies between *MS4A4A* and *MS4A6A* and is associated with increased LOAD risk. The LD between these two SNPs is between 0.4 < r^2^ < 0.6, suggesting they may be having independent effects.

The exact function of these transmembrane proteins is unknown, although they have been implicated in mediating calcium influx, regulating endocytosis, trafficking, and signaling [52], and may act as chemoreceptors [53]. However, their ligand binding partners and downstream signaling cascades have not yet been fully described. High levels of expression of *MS4A6A* in AD brains is associated with increased Braak tangle and plaque scores, indicating advanced disease pathology [30]. While most loci appear to have similar effects in *APOE4* ^*+*^ and *APOE4* ^*−*^ AD patients, the *MS4A* signal is stronger in *APOE4* ^*−*^ subjects [54].

*MS4As* are expressed in microglia and macrophages within the brain, and are also highly expressed in peripheral immune cells. Several of the *MS4A*s, including *MS4A4A* and *MS4A6A*, contain binding regions for the transcription factor PU.1, which is also selectively expressed in myeloid cells and has been implicated in AD. Work by Huang, et al., has shown that changes in expression level of *Spi1,* the gene encoding PU.1 in mouse, correlate with similar expression changes in *Ms4a4a and Ms4a6d* (mouse ortholog to *MS4A6A*) in BV2 microglial cell lines [26]. Further experiments will have to be done to determine the exact function of the *MS4As* and which genes within this family are involved in AD.

### Genes identified through sequencing strategies and rare variants

Sequencing strategies generate high-coverage data of different genetic loci to identify rare variants that are not seen through standard GWAS. Rare variants in two genes, *ABCA7* and *TREM2*, have a strong and replicated association with AD, and are described below.

#### ABCA7

ATP-binding cassette transporter A7 (ABCA7) is a membrane transporter. *ABCA7* is located on chromosome 19p13.3, and common SNPs in this locus have been implicated in LOAD through GWAS. Additionally, this locus contains several rare variants that have been identified by sequencing studies. GWAS in European and African Americans have implicated the chromosomal region including *ABCA7* [17, 24]. Indeed this locus contains the only SNPs to reach genome-wide significance in a GWAS of African Americans apart from those in the APOE locus [17]. One of these SNPs identified in Europeans, rs3764650, is located within an intronic region of *ABCA7*. It is associated with T-box transcription factor binding sites and myeloid-related transcription factors such as CEBPD [33]. The AD-risk SNP identified in African Americans, rs115550680, is also found in an intronic region of *ABCA7* and is in high LD (r^2^ > 0.8) with variants identified in European populations. Both SNPs are associated with weak transcription in the brain and strong transcription in myeloid cells [33].

*ABCA7* is expressed in microglia, and is associated with increased risk of LOAD. ABCA7 is known to transfer phospholipids to apolipoproteins [55], including APOE and APOJ/CLU, and it is possible that mutations in this protein may be affecting AD though this pathway [56–58]. However, *ABCA7* has also been implicated as a modulator of microglial function by its association with microglial phagocytosis and clearance [16]. Multiple rare coding variants in *ABCA7* confer loss of function, resulting in increased risk of AD [59–62]. Indeed, targeted resequencing of *ABCA7* in African Americans with low or high risk alleles at this locus led to the identification of a 44 bp deletion, which results in a frameshift mutation and increased AD risk. However, a recent follow-up study of *ABCA7* mutations challenges the assertion that all of these variants are loss of function, and implicates a role for *ABCA7* in additional neurodegenerative diseases [63].

*ABCA7* has previously been shown to be associated with AD, with increased levels of *ABCA7* correlating to increased plaque burden and more rapid cognitive decline [30, 58]. Increased levels of ABCA7 increase microglial phagocytosis and Aβ clearance, which is believed to be regulated through the C1q complement pathway. Additionally, *ABCA7* ^*−/−*^ mice display increased Aβ deposition, suggesting that this protein is important for the appropriate clearance of Aβ aggregates by microglia [64]. It is possible that ABCA7 can affect AD through multiple pathways, given its multiple roles in transport and microglial function.

#### TREM2

Complete loss of function of TREM2 results in Nasu Hakola syndrome, a rare recessive disorder characterized by multifocal bone cysts and frontotemporal dementia. This is driven by loss of function in macrophages in many tissues, including microglia [65]. TREM2 is a transmembrane-glycoprotein that acts as a receptor on the surface of immune cells of myeloid origin, including microglia, and senses lipids that are exposed after cellular damage. Heterozygous rare variants of *TREM2* are associated with an increased risk of developing AD in European, African American and Asian populations [66–69]. The identified AD-risk SNPs in this region are likely to result in partial loss of function [70]. Evidence suggests that the same variants may increase risk for other neurodegenerative diseases including Parkinson’s Disease (PD), amyotrophic lateral sclerosis (ALS), and frontotemporal lobar degeneration (FTLD) [16, 66, 67, 71]. However, meta-analysis of other diseases shows that data linking *TREM2* to these neurodegenerative diseases are far less compelling than in the AD studies [71].

TREM2 associates with its adaptor protein, TYROBP/DAP12, which is responsible for the propagation of downstream signaling through this receptor [65]. Network analysis of whole transcriptome gene expression data in healthy individuals identified *TREM2* as a central hub in five brain regions, suggesting that it can drive expression levels of its associated genes [72]. Many of the genes that associate with *TREM2* are immune genes, and others have previously been associated with AD risk [72]. *TREM2* expression is increased in AD, which may be a compensatory mechanism, as AD-associated *TREM2* mutations are partial loss of function and can affect receptor binding of TREM2 to its associated ligands [73, 74].

Activation of TREM2 through ligand-receptor binding stimulates macrophage and microglia phagocytosis, survival, and mobilization, whereas absence of TREM2 in microglia impedes their ability to phagocytose and alters their gene expression signatures [75]. TREM2 has been shown to bind apolipoproteins, including APOE and APOJ/CLU, and disease variants of TREM2 showed lower binding to these AD-associated ligands [76]. *Trem2* ^*−/−*^ mice bred to an accelerated AD mouse model (5XFAD transgenic mice) show significant Aß accumulation in hippocampal regions and an increase in insoluble Aß [77]. These mice also show defects in microglial reactivity, Aß plaque clearance, and survival [77, 78]. The selective expression of *TREM2* in microglia, the association of *TREM2* with other immune genes, and the impaired function of *TREM2* mutant microglia suggest that this cell type is a key player in the development in LOAD.

### Additional plausible microglial genes

#### CD33

GWAS studies in multiple ethnic populations have identified variants within the *CD33* locus, located on chromosome 19q13.33, that are associated with AD. These loci were genome-wide significant in early studies, but fell short of genome-wide significance in large meta-analyses [23, 49]. The variant rs3865444 is associated with *CD33* exon 2 splicing [27] and with a reduction in *CD33* expression [79]. Fine-mapping studies have shown that rs12459419, a SNP in high LD with rs3865444, likely mediates the altered splicing [27]. Both variants are associated with chromatin marks signifying low/repressed transcription in brain tissues, but is closely associated with active transcription start sites in myeloid cells. Introduction of rs12459419 into microglial BV2 cells modulates exon 2 splicing efficiency and microglial activation [27].

CD33, a member of the sialic acid-binding Ig-like family, is a myeloid cell receptor that is exclusively expressed by microglia and macrophages in the brain. Increased *CD33* expression is seen in AD brain and is associated with increased plaque burden, advanced cognitive decline, and disease severity [30, 80]. *Cd33* ^−/−^ mouse models indicate that knocking out CD33 results in lower Aß levels and reduced amyloid plaque burden in brain [79]. However, these mice do not exhibit altered APP processing, suggesting that it may be Aß clearance mechanism of microglia that is responsible for this phenotype.

#### *INPP5D/*SHIP1 and *CD2AP*

GWAS have also identified common variants in the locus that codes for phosphatidylinositol-3,4,5-trisphosphate-5-phosphatase 1, also known as SHIP1, which is encoded by the gene *INPP5D*. The AD-risk variant rs35349669 is associated with high gene expression in myeloid cells, and weak expression in brain tissues and neurons. It is near another gene *CD2AP*, which was also identified in GWAS by the SNP rs10948363. However, this SNP is associated with low transcription in both brain and myeloid cells.

*INPP5D* and *CD2AP* are both expressed in macrophages and microglia within the brain. SHIP1 is linked to another major AD risk gene TREM2, and inhibits TREM2 signaling through the necessary adaptor DAP12 [81]. *INPP5D* has also been identified as associated with AD in network analysis, providing additional support for its involvement in AD [41]. These data suggest that *INPP5D* may be playing a relevant role in microglia in AD.

## Gene expression networks in AD

Insights as to the contribution of microglia in AD have been uncovered through gene expression network analysis. By comparing gene expression changes between healthy and AD samples, we can identify quantifiable internal cellular changes, assess the contributions of these genes, and hypothesize functional interactions. There are two methods by which we can approach gene expression analysis to understand AD etiology; the first is through a candidate gene approach, by identifying genes of interest and looking at their co-expression partners. The second is by constructing network modules based on functional associations between genes and looking for enrichment of these modules in disease states.

Most AD-associated risk variants identified through GWAS have not been related to gene expression changes in brain whole brain tissue [82]. That is, most of these risk loci do not overlap with brain eQTLs. In many cases, AD-risk genes identified through GWAS are expressed at very low levels in brain samples [82], which limits our ability to study these genes further. These low expression signatures may be because the heterogeneous population of brain cells sampled for these studies dilutes cell-specific signatures of small populations of cells, including microglia. By looking at co-expression networks and cell-specific signatures, we can compensate for the low expression of microglial genes in brain (as compared to the gene expression seen in other brain cell types) and gain insight as to the roles of these genes to determine their contribution to AD pathology.

Gene expression data networks can be used to identify a gene’s interaction partners and subsequently, the cellular systems that are dysregulated in disease. Building these networks will teach us more about their normal function and identify targets for future therapies. Previous work in whole-brain gene expression datasets has narrowed down network signatures to identify microglial dysfunction as a key element of AD [41, 72, 83]. These analyses revealed AD-associated networks as involved in innate and adaptive immunity, providing support for the role of microglia and/or brain infiltrating peripheral myeloid cells in AD.

### Interaction of key AD drivers in healthy controls

One such method for conducting network analysis is to perform weighted gene co-expression network analysis (WGCNA), which groups genes into association modules in an unsupervised manner, such that genes within these modules are co-expressed and co-regulated. The shared regulation of these genes suggests that they are also functionally related. Forabosco et al. conducted a WGCNA, in which they analyzed microarray gene expression data from ten brain regions of 101 pathologically normal individuals [72]. They then grouped genes into association modules, focusing on *TREM2*, a major risk factor for LOAD that has also been identified in GWAS [84]. They discovered that *TREM2* is a central and highly connected gene within its expression module, suggesting that it is a hub that drives module function. The *TREM2* module shares a significant number of genes with another microglial module, and is enriched for genes of the innate immune system and adaptive immune system, such as the microglial-specific genes *CX3CR1, ITGAM, AIF1, FCER1G,* and *CD68*. The authors then looked at a number of other genes that have previously been associated with AD through additional studies, including GWAS. Strikingly, the *TREM2* module is also enriched for genes implicated in AD, suggesting that under normal physiological conditions AD-risk genes interact to promote normal function of microglia. As network analysis evolves, newer and improved techniques such as multiscale embedded gene co-expression network analysis (MEGENA) can also be used to verify these findings [85].

### Co-expression networks in brains of AD-affected individuals

Zhang et al., conducted a similar analysis with case and control samples from three brain areas in 376 LOAD patients and 173 non-demented controls [83]. They then constructed multi-tissue co-expression networks to investigate the differences in these modules between case and control samples. The number of modules differed between cases and controls, and new modules were created (described as “gain of connectivity”) and existing modules were disrupted (described as “loss of connectivity”) in AD. They then ranked the modules through an integrative network-based approach that looked at module disease association, based on clinical and neuropathological findings and network properties. In such a ranking, the highest ranked module would contain genes most highly associated with disease. Highest ranked was the immune/microglia module, which was a gain of connectivity module that showed the most functional enrichment in this analysis. It is possible that the decreased rank of neuronal modules and increased rank of glial modules may be due to the depletion of neurons within AD brains. However, the role of an immune component in AD was supported by further interrogation of this module. *TYROBP*, also known as DAP12, and the key adaptor protein for the function of TREM2, was determined to be a key driver in this module. Together, these data support the role of *TREM2* and its associated immune-related signaling partners in AD.

### Epigenetic signals in AD suggest immune component

Previous gene expression network papers have highlighted the important role of *TREM2* and its associated immune networks as seen through expression network analysis. However, these investigations did not look closely at epigenetic regulation of gene expression. Gjoneska et al. sought to investigate the contribution of epigenetic factors to neurodegeneration using an AD mouse model (CK-p25) at early and late stages of disease, as well as postmortem brain samples from 22 AD patients [41]. In their transcriptome analysis, they observed consistent enrichments in immune-related genes, similar to what was seen in previous experiments. In their epigenome analysis, they used ChIP-seq to profile seven chromatin marks associated with both activation and repression of transcription. They identified molecular signatures associated with AD, including depletion of neuronal promoters and enhancers, and an enrichment of AD-associated loci in enhancer regions. They also identified a putative therapeutic target, PU.1 (coded for by the gene *SPI1)* a transcription factor that is associated with microglial activation and immune function, and whose binding motif was enriched in their analysis. As with previous network analyses, it is possible that the enrichment of these epigenome marks associated with microglia could reflect the increased presence of microglia and fewer neurons (as a result of neurodegeneration) in AD brains, rather than changes within the cell. However, follow-up functional studies support the role of immune-related genes, including *SPI1*, in AD [26].

Together, these network analyses from brain tissue suggest that AD-associated genes are co-expressed and co-regulated under normal conditions to support the normal function of microglia in the brain. Under disease conditions, these networks can be disrupted and expanded, and such disruptions are correlated to the presence and severity of AD. Through looking at additional factors such as gene regulation, we can validate the contribution of important gene targets such as *TREM2* and *SPI1*, as well as identify novel targets associated with AD.

## Conclusion

### Modeling microglial involvement in AD

Genes identified by GWAS and sequencing, as well as expression network analyses support the involvement of microglia in the development and progression of AD. However, it is still not understood how all of these genes functionally interact to promote disease pathology and whether or how they contribute to the prevailing amyloid cascade hypothesis.

The majority of studies of microglial involvement in AD have focused on one particular function: microglial clearance of Aß plaques. It is suggested that when the natural mechanisms that regulate the phagocytic function of these cells are disrupted, Aß accumulates and activates the cascade that promotes subsequent neuronal degeneration [86]. This is supported by network analyses, which show that normal microglial networks are disrupted in AD. *CD33*, *ABCA7*, and *TREM2* have all been implicated in microglial phagocytosis. It is also supported by many functional studies, which show microglial clearance of Aß plaques is disrupted in AD mouse models.

However, while Aß plaques are a defining feature of AD, they are not the only feature, and it is possible that microglia have other underexplored related functions. Microglia display increased proliferation in LOAD, which is mediated by the receptor colony-stimulating factor 1 (CSF1R) [87]. Microglial proliferation correlates with disease severity in humans and in transgenic mouse models of AD [87]. Inhibiting CSF1R in AD mouse models (including APPswe [88], PSEN1dE9 [89], APP/PS1 [88], and 5xfAD mice [90]) decreases the proliferation of microglia and rescues behavioral deficits [87, 91]. However, the number of Aß plaques is not significantly changed, suggesting that their presence is not directly contributing to AD progression. This is in contrast to previous studies, which focus on microglial engulfment of Aß as a major indicator of disease impact [92]. The importance of microglial proliferation and activation is also supported by *APOE* isoform differences in microglial response and migration. This, in turn, can impact clearance of Aß or influence other functions [93, 94].

Microglia may also have other clearance targets beyond Aß plaques. The significance of *APOE* and *TREM2* on LOAD risk suggests that lipoproteins may be playing a larger role in this pathway than previously anticipated, and that lipid-sensing features of microglia contribute to disease. It is possible that microglia are specifically tagging similar lipid-rich cellular debris for phagocytosis. This theory is supported by initial reports of the disease made by Alois Alzheimer in 1907, who described the accumulation of “adipose saccules,” or “lipid droplets,” within glial cells. Lipid droplets are lipid-rich organelles that also contain cholesterol and triglycerides, and the accumulation of these droplets has been linked to cellular stress and neurodegenerative diseases including ALS and AD [95]. Mouse models of AD amyloidosis (Tg2576) show increased lipid peroxidation, which precedes amyloid plaque formation [96]. It is possible that accumulation of these lipids and associated oxidative damage contribute to AD pathology. Whether these lipid droplets are causative or reflective of abnormal glial function remains to be investigated.

**Fig. 2:**
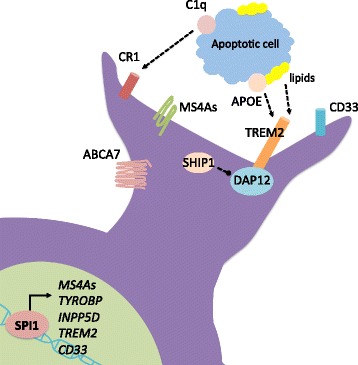
Efferocytosis model of microglia in LOAD. Microglia contain multiple receptors that can recognize cellular distress signals and other ligands on apoptotic or necrotic cells, including lipids, C1q, and APOE. Propagation of downstream signals activate the transcription factor PU.1, which transcribes the majority of the genes involved in these pathways

The enrichment of immune pathways in AD is complemented by the enrichment of other pathways, including endocytosis and cholesterol metabolism. These three pathways involve many of the same genes, including (but not limited to) *APOE*, *TREM2*, and *ABCA7*. Combining these pathways together suggests a new model of microglial involvement in AD relating to microglial efferocytosis, or clearance of dying cells. The efferocytosis model reveals a functional role for microglia and support for the enrichment of immune genes in AD that has been revealed through gene network studies and disease genetics (Fig. 2). Many of these genes interact either directly (as with binding partners APOE and TREM2) or indirectly (as with PU.1 and its downstream gene targets). It is possible that these three pathways converge within microglia, which require an ability to respond, engulf, remove, and process cellular debris.

### What confers risk: endogenous microglia or invading peripheral immune cells?

As indicated previously, microglia share a similar developmental pattern, gene expression, and function with other immune cells. It is possible that other myeloid cells, such as monocytes and macrophages, could be contributing to the observed immune effect. The strong association between LOAD signals and myeloid cells provides evidence for the immune system’s role in LOAD, and this relationship can be exploited to investigate genetic risk factors and potentially use peripheral immune cells as a source for easily accessible biomarkers to monitor, diagnose, and eventually treat LOAD. However, another interpretation of these data suggests that the relevant cell-types could be residing outside of the brain, and that the relationship between LOAD and the immune system is controlled by infiltrating peripheral cells [28].

Deciphering the exact contribution of resident microglia versus infiltrating peripheral immune cells in LOAD will be of utmost importance. There are unique molecular and functional signatures in microglia that are not retained in microglial cell lines and are not observed in monocytes that are recruited to the central nervous system, which will serve as a useful tool in determining the contribution of each [97]. Similarly, microglia and infiltrating monocytes have different functions. Monocyte-derived macrophages are highly phagocytic and inflammatory, and can initiate demyelination of neurons [98]. In contrast, microglia have a suppressed cellular metabolism and perform more surveying functions and clear debris [98]. It will take time to determine which of these pathways is contributing most to LOAD.

Genetic studies play an essential role in identifying the molecular mechanism of disease, particularly in highly heritable diseases such as AD. These studies, conducted in mouse models and human subjects, have uncovered the role of microglia in disease and suggest that this cell type plays an active role in AD pathogenesis. Further studies will need to be conducted in order to confirm whether the immune component of AD is from resident tissue macrophages such as microglia, or due to the infiltration and activation of peripheral immune cells into the brain. However, this new area of study updates our understanding of AD, and provides a wider range of targets for drug discovery and development.

## Abbreviations

AD: Alzheimer’s disease
ADAD: Autosomal dominant Alzheimer’s disease
ALS: Amyotrophic lateral sclerosis
ChIP-seq: Chromatin immunoprecipitation and sequencing
EOAD: Early onset Alzheimer’s disease
eQTL: expression quantitative trait loci
FTLD: Frontotemporal lobar degeneration
GWAS: Genome-wide association study
GWAX: Genome-wise association study by proxy
IGAP: International Genomics of Alzheimer’s Project
LD: Linkage disequilibrium
LOAD: Late onset Alzheimer’s disease
MEGENA: Multiscale embedded gene co-expression network analysis
PD: Parkinson’s disease
SNP: Single nucleotide polymorphism
WGCNA: Weighted gene co-expression network analysis
WGS: Whole genome sequencing
